# Self‐Healing UPy‐Functionalized Polyethylene Networks: A Robust Platform for Resilient and High‐Performance Lithium Battery Separators

**DOI:** 10.1002/advs.77850

**Published:** 2026-09-27

**Authors:** Daniele Callegari, Arkadiusz Zych, Roberta Pinalli, Enrico Dalcanale, Eliana Quartarone

**Affiliations:** ^1^ Department of Chemistry and INSTM‐GISEL University of Pavia Pavia Italy; ^2^ Department of Chemistry Life Sciences and Environmental Sustainability and INSTM University Of Parma Parco Area delle Scienze Parma Italy

**Keywords:** autonomous self‐healing, battery safety, hydrogen bonds, lithium‐ion batteries, polymers

## Abstract

Conventional polyolefin separators exhibit limited mechanical resilience and poor damage tolerance, leading to performance degradation and critical safety concerns. To overcome these limitations, we developed supramolecular self‐healing separators based on polyethylene–hydroxyethyl methacrylate (PE‐HEMA) copolymers functionalized with ureidopyrimidinone (UPy) units. The UPy motifs undergo reversible dimerization through quadruple hydrogen bonding within the polymer matrix, forming a dynamic supramolecular network capable of autonomously repairing mechanical defects. Three copolymers with varying UPy grafting densities were synthesized and blended with high‐molecular‐weight polyethylene oxide (PEO) to significantly enhance electrolyte affinity and ionic transport. Composite membranes (35–40 µm) were subsequently produced via a rapid, solvent‐free hot‐pressing process. Due to increased amorphous character and enhanced polymer chain mobility, the resulting materials exhibit efficient self‐healing at mild temperatures (40°C), improved electrolyte wettability, and high ionic conductivity. Electrochemical testing in Li|Li symmetric cells demonstrate that the separators successfully recover functionality after dendrite‐induced short circuits through network reorganization. When implemented in LiFePO_4_‐based full cells, the optimized separator enables stable cycling, delivering a discharge capacity of ≈130 mAh g^−^^1^ after 1000 cycles with up to 81.5% capacity retention, alongside improved thermal safety. These results highlight the promise of UPy‐based supramolecular separators for next‐generation lithium‐based batteries.

## Graphical abstract

Self‐healing separators are developed by grafting ureidopyrimidinone (UPy) quadruple hydrogen‐bonding motifs onto PE‐HEMA copolymers, blended with high‐MW PEO via solvent‐free hot‐pressing. The dynamic UPy network enables autonomous healing, restoring functionality after short circuits. The membranes exhibit improved electrolyte affinity, enhanced ionic conductivity, superior LFP cell stability, and increased thermal safety under abuse.

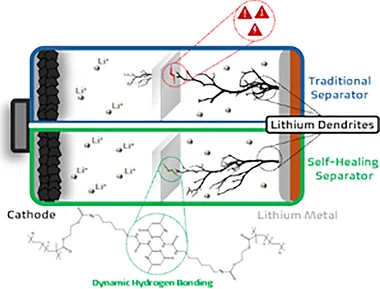

## 1 Introduction

Lithium‐ion batteries (LIBs) dominate the current landscape of portable electronics, electric vehicles, and grid‐scale energy storage owing to their high energy density and long cycle life [1, 2]. Although substantial progress has been achieved in electrode and electrolyte engineering, the separator, despite its electrochemical passivity, plays a pivotal role in determining the overall performance, stability, and safety of LIBs [3, 4]. Conventional polyolefin‐based separators typically exhibit inadequate thermal resistance and limited mechanical robustness, which can accelerate degradation, promote lithium dendrite formation, and compromise device reliability under demanding operating conditions. Overcoming these intrinsic limitations requires separators that transcend a passive barrier function and instead embody smart, adaptive materials endowed with active safety and reliability features [5, 6, 7].

Looking forward, the integration of smart functionalities represents a paradigm shift in energy‐storage design. Beyond their traditional role, separators can be engineered to exhibit self‐healing, self‐reporting, and self‐regulating behaviors that dynamically respond to operational stress. Self‐reporting capabilities enable early detection of microcracks, thermal hotspots, or chemical imbalances, allowing intervention before catastrophic failure [8]. Self‐regulating features, such as thermally responsive shutdown or ionic‐flux modulation, enhance safety under overcharge, high‐rate cycling, or mechanical abuse [9]. Additionally, emerging scavenger functionalities provide an extra layer of protection by selectively capturing harmful species such as hydrofluoric acid, transition‐metal ions, or radical intermediates generated during electrolyte decomposition, thereby mitigating parasitic reactions and extending battery lifetime [10, 11]. Through the integration of these adaptive behaviors, separators evolve from passive physical barriers into intelligent, multifunctional components that directly contribute to battery performance, safety, and sustainability. This vision aligns closely with the Battery 2030+ roadmap, which identifies smart materials, self‐healing chemistries, and reactive scavenger systems as key enablers for ultra‐durable, safe, and sustainable European battery technologies [12]. Embedding such functionalities at both the molecular and macroscopic scales is expected to play a central role in shaping the next generation of high‐performance electrochemical energy‐storage systems.

Among the various strategies under exploration, self‐healing polymers have emerged as a particularly versatile materials platform capable of autonomously restoring structural integrity and extending device functionality [13, 14, 15]. These materials rely on reversible dynamic interactions including: hydrogen bonding, metal–ligand coordination, dynamic covalent chemistry, and host–guest interactions to repair microcracks and maintain mechanical cohesion. In LIBs, self‐healing behavior directly addresses critical failure modes such as dendrite penetration, separator fracture, electrolyte leakage, and thermal shrinkage [16, 17]. Importantly, self‐healing materials enhance resilience not only under mechanical abuse but also during long‐term cycling and high‐rate operation, conditions that often induce premature failure in conventional separators. Recent advances further demonstrate that combining multiple dynamic interactions or incorporating multifunctional additives can finely tune self‐healing kinetics, mechanical robustness, and electrochemical stability, offering unprecedented control over battery reliability [18].

Incorporating complementary multiple hydrogen‐bonding units has proven effective in imparting mechanical strength and self‐healing capability to pristine polyethylene (PE) [19]. Among the various multiple hydrogen‐bonding motifs, ureidopyrimidinone (UPy) stands out as the most prominent, owing to its strong self‐dimerizing quadruple hydrogen bonds, high association constant, synthetic accessibility, and availability of functional derivatives for polymer modification [20].

Here, we report a supramolecular self‐healing separator (SHS) based on a polyethylene/hydroxyethyl methacrylate (PE‐HEMA) copolymer obtained via high‐pressure radical copolymerization of ethylene with HEMA [21], functionalized with UPy units grafted onto the HEMA moieties [19]. The UPy groups form dynamic quadruple hydrogen‐bonded dimers within the polymer matrix, enabling autonomous repair. Three polymer variants, P1, P2, and P3, were synthesized to investigate the influence of HEMA content and UPy incorporation on network cross‐linking, thermal stability, mechanical resilience, and electrochemical performance. Increasing UPy content enhances cross‐link density, providing tunable structural and functional properties. Blending these UPy‐functionalized PE‐HEMA copolymers with high‐molecular‐weight polyethylene oxide (PEO, Mw ≈ 300 kDa) further improves mechanical strength, electrolyte affinity, and ionic transport. The resulting hybrid separators combine the adaptability of supramolecular networks with the tunability of polymer blending, offering a robust platform for next‐generation self‐healing separators. Overall, this study demonstrates that the strategic integration of dynamic bonding motifs into separator architectures enables precise control over mechanical, thermal, and electrochemical properties.

## 2 Results and Discussion

### 2.1 Design of the Self‐Healing Polymers PE‐HEMA‐UPy

**SCHEME 1:**
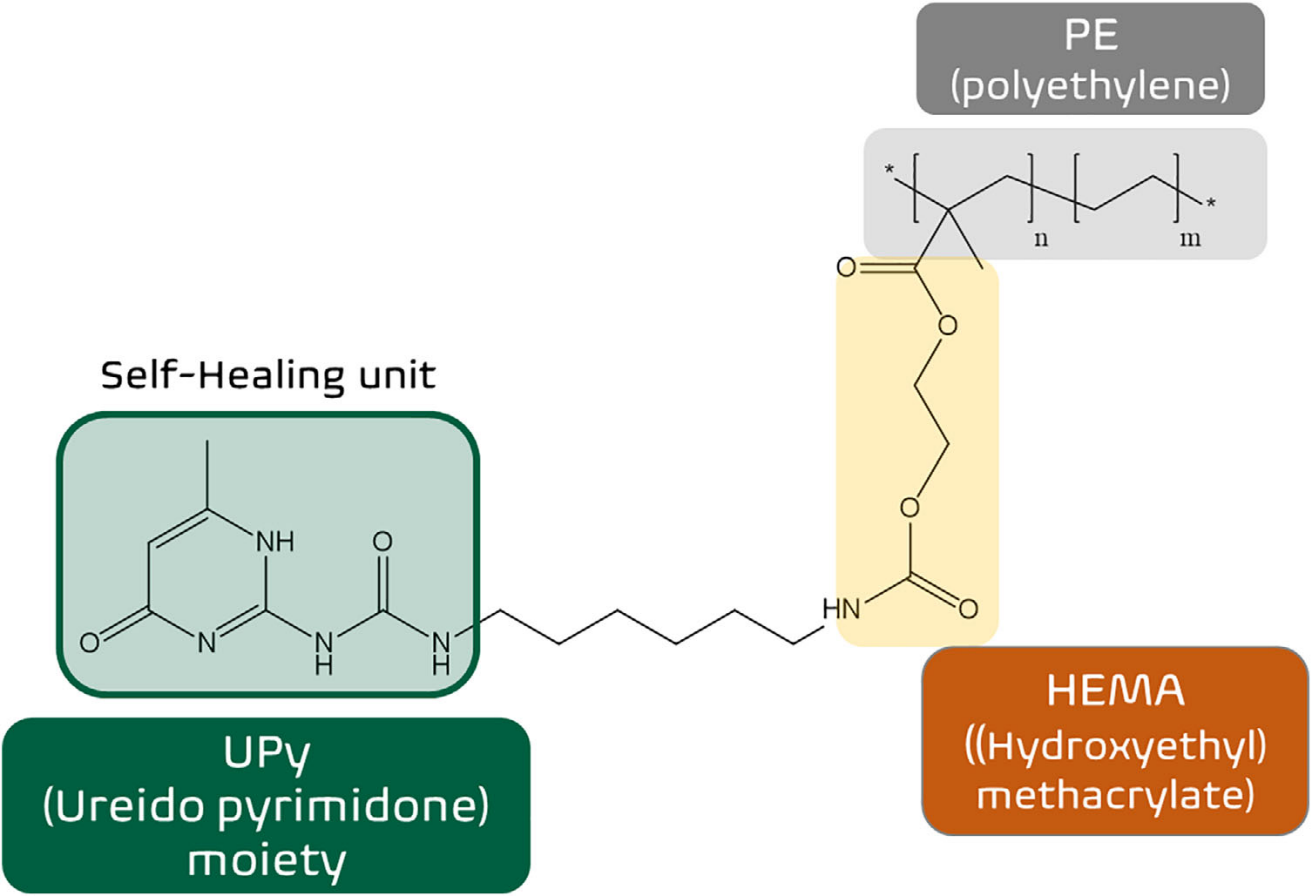
Schematic representation of the realized self‐healing polymer based on a polyethylene (PE) backbone functionalized with ureido‐pyrimidinone (UPy) moieties via (2‐hydroxyethyl) methacrylate (HEMA) segments.

A copolymer matrix was properly designed by connecting three different blocks as functionalizing units to confer self‐healing capability to innovative separators for next generation LIBs (as reported in Scheme 1). The PE‐HEMA‐UPy self‐healing polymer architecture comprising three complementary components with distinct roles:

i) The polyethylene (PE) backbone constitutes the primary structural matrix of the system, providing mechanical strength, toughness, and overall dimensional stability. This phase ensures load‐bearing capacity and effective stress distribution throughout the material, serving as a robust framework within which the dynamic functionalities are embedded.
ii) The (2‐hydroxyethyl) methacrylate (HEMA)‐derived segments act as flexible linkers that connect the functional moieties to the PE backbone. These segments enhance chain mobility and introduce sufficient segmental flexibility, enabling local rearrangements of the polymer chains. This mobility is crucial for facilitating the reorganization of the supramolecular interactions following mechanical damage.
iii) The ureido‐pyrimidinone (UPy) moieties function as dynamic supramolecular units capable of forming strong and reversible quadruple hydrogen bonds. These interactions are responsible for the autonomous self‐healing behavior of the material, as they can reversibly dissociate under stress and subsequently re‐form when damaged interfaces are brought back into contact, thereby restoring structural integrity. The UPy grafting onto functionalized PE‐HEMA was achieved via formation of urethane links between the UPy isocyanate precursor and the primary hydroxyl groups of the HEMA unit.

Three different PE‐HEMA‐UPy polymers were selected for the present work. They differ for the degree of HEMA content in the copolymer and for the degree of UPy functionalization (Table 1). The PE‐HEMA copolymers were prepared via high pressure copolymerization of ethylene and 2‐hydroxyethyl methacrylate in an autoclave, with a peroxide initiator, following a published procedure [21]. The solution grafting procedure consists of dissolving the PE‐HEMA in toluene with the subsequent addition of UPy isocyanate and the dibutyltin dilaurate (DBTDL) catalyst [19].

**TABLE 1:** Above: Composition and physicochemical properties of PE‐HEMA copolymers including HEMA content (mol%), number‐average molecular weight (M_n_), weight‐average molecular weight (M_w_), dispersity (Đ), melting temperature (T_m_), and β‐relaxation temperature (T_β_). Below: Grafting efficiency and thermal properties of UPy‐functionalized polymer samples, including percentage of UPy grafting, UPy content (mol%), melting temperature (T_m_), β‐relaxation temperature (T_β_) and degree of crystallinity (X_C_).(a) det. via ^1^H NMR and N elemental analysis (Figures S9–S11 and Table S3); (b) det. via GPC; (c) decreasing molecular weight due to HEMA acting as chain transfer agent [22]; (d) det. via DSC; (e) det. via DMTA; (f) calculated with heat of fusion of PE = 286.2 J/g [23].

Sample	HEMA (mol%)^a^	Mn^b,c^ (g/mol)	Mw^b,c^ (g/mol)	Ð^b^	Tm^d^ (°C)	Tβ^e^ (°C)
PE‐HEMA 1	0.3	17800	62000	3.5	112.9	10.0
PE‐HEMA 2	0.8	16200	63300	3.9	109.0	8.8
PE‐HEMA 3	4.2	4000	16900	4.2	98.4	−3.5
Sample	% UPy grafting^a^	UPy (mol%)	Tm^d^ (°C)	Tβ^e^ (°C)	X_C_^d,f^
P1	75	0.22	110.8	36.0	40.3
P2	65	0.52	102.9	33.2	28.4
P3	30	1.26	88.6	21.5	13.7

### 2.2 Self‐Healing Separators

#### 2.2.1 Blending PE‐HEMA‐UPy with PEO

Self‐Healing Separators (SHSs) were prepared via a rapid, solvent‐free and scalable hot‐press method, by premixing PE‐HEMA‐UPy with high‐molecular weight polyethylene oxide (PEO 300kDa), in a 2:1 weight‐to‐weight (w/w) ratio, in order to favor higher electrolyte uptake and ionic affinity (see Figure S1). Hot pressing was performed at 120°C and 400 psi for 10 min, ensuring polymer softening and phase interdiffusion without any thermal degradation, as properly described in the experimental section. SEM images in Figure S2 show that the resulting films exhibit self‐standing properties with compact and uniform texture and thickness values in the range 35–40 µm, compatible for applications in lithium‐ion battery [24, 25].

Although the blending step does not involve any solvent, the two components are combined by exploiting their molten state rather than their mutual solubility, which makes the procedure essentially not dependable to the high molecular weight of the PEO. Before hot pressing, PE‐HEMA‐UPy and PEO were grounded and mechanically premixed as fine powders, so that the diffusion length required in the subsequent mel step is reduced to the sub‐millimeter scale. The pressing temperature (120°C) lies above the melting point of the crystalline PEO phase (≈68°C) and the PE‐HEMA‐UPy matrix (88.6–110.8°C, as reported in Table 1). Under these conditions the applied pressure (400 psi for 10 min) forces the two molten polymers to flow, spread and interdiffuse, the high melt viscosity associated with the large molecular weight of the PEO (300 kDa) being compensated by the long residence time and by the shear imposed during the pressing stage. The chemical integrity of the functional groups after the thermal treatment, and the occurrence of specific interaction between the two blend components, were investigated by means of infra‐red spectroscopy.

**FIGURE 1:**
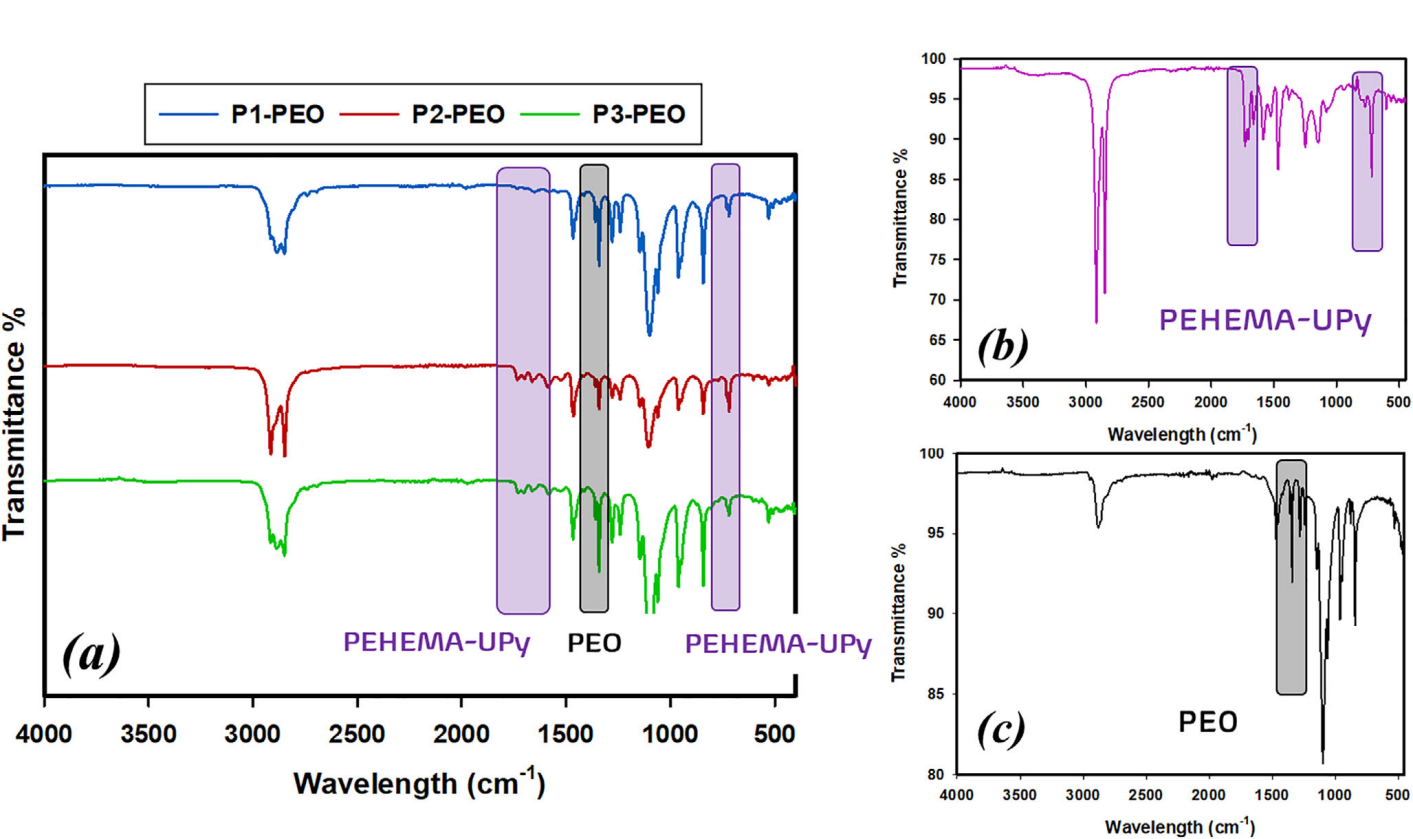
(a) FTIR spectra of the self‐healing separators. Spectra of the precursors: (b) PE‐HEMA‐Upy and (c) PEO300kDa.

Figure 1 shows the FTIR spectra of polymeric separators P1‐PEO, P2‐PEO, and P3‐PEO. Specifically, in Figure 1a highlights that all samples exhibit the main characteristic signals of the constituent components. The presence of the ureido‐pyrimidinone (UPy) group is confirmed by the bands at 1669 cm^−1^ (ρ CN, ρ CC), 1588 cm^−1^ (δ NH) and 1529 cm^−1^ (δ NH) that can be associated with the quadrupole hydrogen bonds. Furthermore, the chemical bonding between the HEMA and the UPy moiety is confirmed by the peak at 1699 cm^−1^ (ν CO), which can be attributed to the formation of the urethane linkage [26, 27]. The bands highlighted in purple (in Figure 1a,b) correspond to the functional groups of PE‐HEMA‐UPy, while those in grey (Figure 1a,c) are attributable to PEO. A strong band around 1720 cm^−1^, corresponding to the carbonyl (C = O) bond of PE‐HEMA‐UPy, is observed in all samples. Signals in the 1100–1300 cm^−1^ region, characteristic of the C─O─C bonds of PEO, are especially evident in the pure PEO sample (Figure 1c) and are present in the blends with varying intensity, indicating good interaction between the two polymers. Additionally, bands in the 3300–3500 cm^−^^1^ region, attributed to N─H and O─H bonds of PE‐HEMA‐UPy, show slight shifts in the blends, suggesting the formation of hydrogen bonding interactions between PE‐HEMA‐UPy and PEO. Overall, the spectra confirm that the polymeric separators effectively combine the structural features of both polymers without loss of the main functional groups, supporting the formation of an integrated, potentially self‐healing blend material. It should be highlighted, however, that infra‐red spectroscopy probes the local environment of the functional groups and, as such, cannot by itself provide information on the phase behavior of the blend, which is instead addressed by the morphological (Figure S2) and thermal (Figure S3) analyses reported.

Figure S3a,b compares the thermogravimetric behavior of the pristine polymers with the composite separators. As shown in Figure S3a, neat PEO300k undergoes a rapid weight loss starting at ≈250°C, indicating limited thermal stability, whereas the PE‐HEMA‐UPy systems (P1, P2, and P3) exhibit a significantly higher degradation onset (≈380  C–400°C) and decompose completely near 480°C–490°C. Despite the high fraction of PEO in the blends, all the separators (Figure S3b) remain stable up to 350°C. This improved thermal stability is ascribed to the formation of a supramolecular network that hinders the chain mobility and delays thermal decomposition.

#### 2.2.2 Separator Self‐Healing capability

**FIGURE 2:**
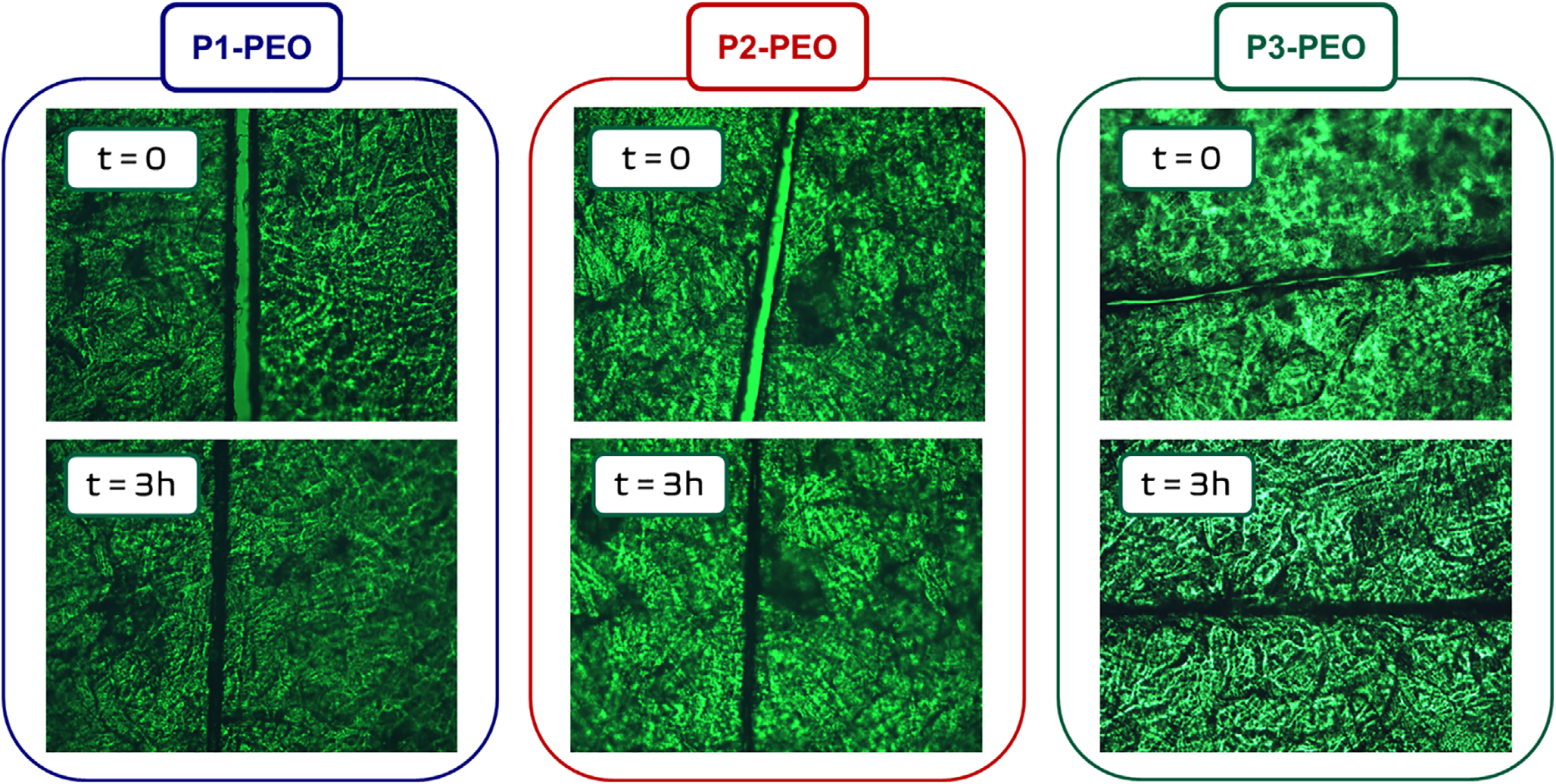
Optical microscopy images of the (a) P1‐PEO, (b) P2‐PEO, and (c) P3‐PEO separators before and after self‐healing at 40°C.

The self‐healing capability of the supramolecular polymer‐based separators was systematically evaluated by forming a deep and sharply defined notch on the membrane surface with a scalpel and subsequently monitoring the recovery at 40°C, as reported in Figure 2. At t = 0, all samples display a sharp and continuous incision, clearly distinguishable from the surrounding polymeric matrix. After 3 hours, a remarkable reduction of the damaged area is observed in each case, with the notch almost completely healed. This behavior demonstrates the self‐healing nature of the designed supramolecular network, enabled by the presence of reversible UPy–UPy hydrogen‐bonding motifs that promote dynamic reorganization and reformation of the polymer structure [28]. In contrast, a separator based on PE–HEMA–PEO (Figure S4) did not exhibit any detectable self‐healing after damage, with no structural recovery observed even after 3 or 18 hours, further highlighting the crucial role of UPy‐mediated supramolecular interactions to enable the healing process.

**FIGURE 3:**
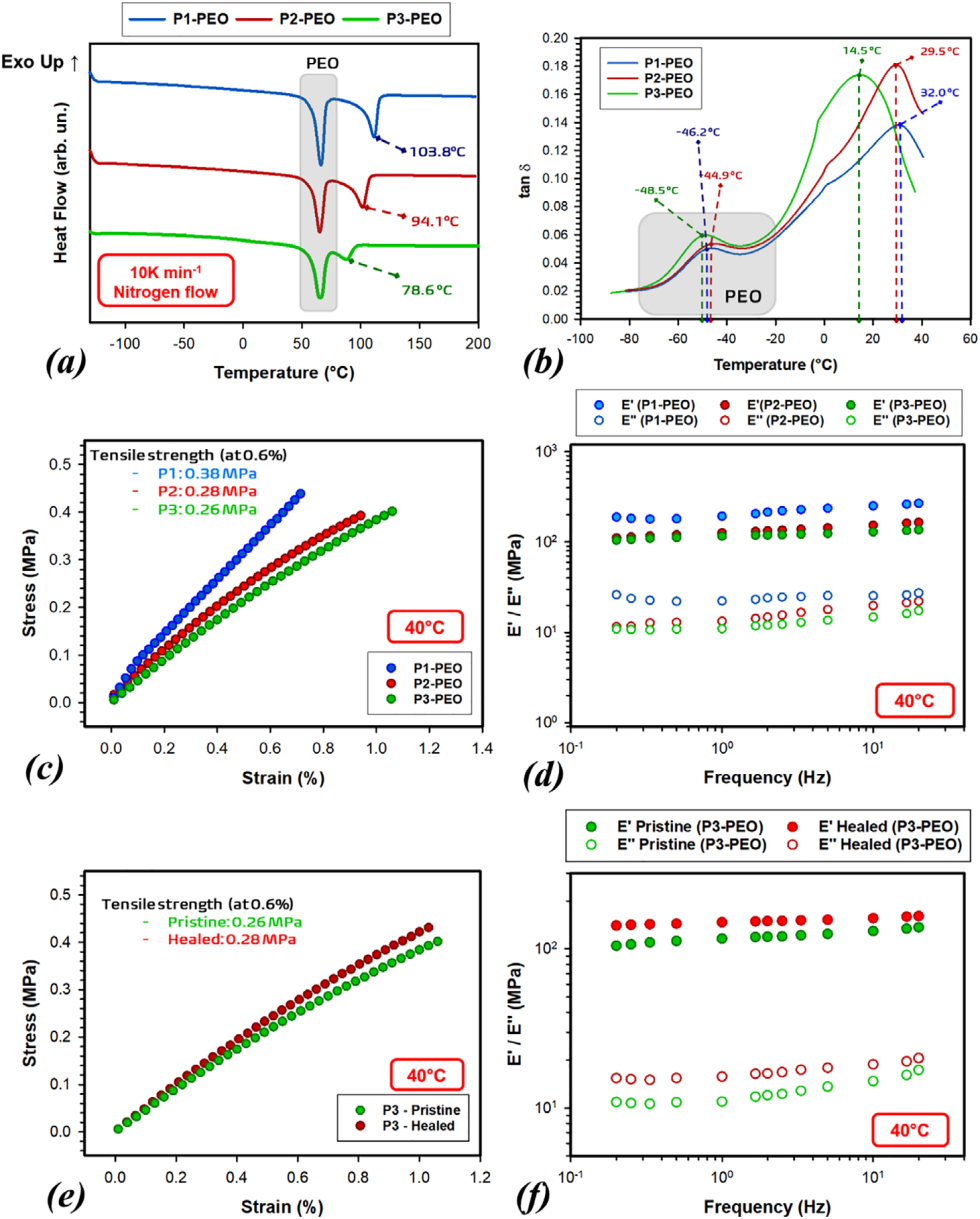
Characterization of PE‐HEMA‐UPy – based separators. (a) DSC thermograms from ‐130°C to 200°C, at a heating rate of 10 K min^−1^. Dynamic mechanical analysis: (b) Evaluation of the beta (β) transition temperature (T_β_) by means of analysis of the tand plots vs T; (c) Stress‐strain plots at 40°C and (d) Frequency sweep tests. (e, f) Comparison between Pristine and Healed P3‐PEO by means of stress‐strain and frequency sweep tests, respectively.

The efficient self‐healing capability of the separators is the consequence of the robustness of the supramolecular interactions combined to the PEO chain dynamics. The addition of high‐molecular‐weight PEO, in fact, provides enhanced segmental mobility, which facilitates chain rearrangement and accelerates the healing process [29]. The different formulations exhibit comparable healing efficiency, indicating that the hot‐pressing fabrication method yields compact and homogeneous membranes capable of effectively exploiting supramolecular dynamics under mild thermal conditions. This self‐healing behavior is particularly beneficial for lithium‐ion battery separators, where mechanical damages can impair ionic conductivity, generate local hot spots, or even trigger short circuits [30]. Remarkably, this efficiency is preserved across all self‐healing polymer compositions, even in the P1 formulation, despite its significantly lower content of UPy moieties. Specifically, P1 (0.3 mol % HEMA, 0.22 UPy mol %), P2 (0.8 mol % HEMA, 0.52 UPy mol%), and P3 (4.2 mol % HEMA, 1.26 UPy mol%, see Table 1) demonstrated similar self‐healing performances. These results underline that efficient self‐repair can be achieved even with low HEMA content and partial UPy functionalization, highlighting the robustness and tunability of the supramolecular approach. A molecular‐level rationale of the healing process can be built on three independent pieces of evidence. First, the repair is completely suppressed in the UPy‐free PE‐HEMA‐PEO control membrane (see Figure S4), which shares the same composition, molecular weight range and processing history: the UPy‐UPy quadruple hydrogen bonds are therefore a necessary condition for the healing, and no contribution from chain interdiffusion or from PEO phase alone can account for it. Second, the FTIR spectra of the separators (reported in Figure 1a) shown the vibrational fingerprint for the dimerized UPy motif, namely the bands at 1669, 1588, and 1529 cm^−1^ arising from the hydrogen‐bonded C═ N/C═C and N‐H moieties, which confirms that the UPy units are effectively engaged in quadruple hydrogen bonds in the as‐prepared membranes and are not merely dispersed as isolated groups. Third, the healing is performed at 40°C, above the β relaxation of the copolymer matrix (T*_β_* = 14.5°C–32°C, see Figure 3b): this is precisely in the regime in which the segmental mobility needed to bring the fractured surfaces into contact and to allow the dissociation and subsequent re‐association of the UPy dimers becomes thermally activated.

#### 2.2.3 Thermal and Mechanical Properties of the Self‐Healing Separators

Figure 3 summarizes the thermal and mechanical characterization of the three separators. Differential scanning calorimetry (DSC) profiles (Figure 3a) show two distinct endothermic transitions. The first peak, highlighted by the grey box, corresponds to the melting of PEO crystalline part and appears at approximately 68°C for all compositions. A second endothermic phenomenon at higher temperature is ascribed to the P1‐P2‐P3 polymer matrix. Notably, moving from P1 to P3, the melting peak progressively decreases in intensity and becomes increasingly asymmetric, suggesting a reduction of the crystallinity degree. This trend is quantitatively confirmed by the melting enthalpies, which decrease from −34.46 J g^−^^1^ (P1) to −27.84 J g^−^^1^ (P2) and −10.3 J g^−^^1^ (P3). β transition temperatures, *T_β_*, determined by means of Dynamic Mechanical Analysis (DMA) (Figure 3b), further support this behavior. Two relaxation processes are observed within the investigated temperature window (−80°C to 40°C): one at low temperature between (−48°C and −45°C), assigned to the cooperative segmental motion of the amorphous PEO phase, and a second at higher temperature, at 14.5°C (P3), 29.5°C (P2), and 32°C (P1), arising from the cooperative segmental motion of the amorphous fraction of the PE‐HEMA‐UPy copolymer, further restrained by the physical cross‐links provided by the UPy dimers. Both processes are therefore glass‐transition‐like relaxation of the amorphous phases and are not to be intended as motions of the chain backbone. Following the nomenclature commonly adopted for polyethylene and its copolymers, the relaxation associated with the segmental dynamics of the amorphous phase in conventionally labelled as the β relaxation (T*_β_*), and this notation is retained here for consistency with the previous characterization of the PE‐HEMA and PE‐HEMA‐UPy systems. The α relaxation of polyethylene, which is related to chain motions within the crystalline lamellae and at the crystal‐amorphous interface, occurs at high temperature and therefore falls outside the temperature range explored (see Experimental Section in Supporting Information). The shift of these relaxations to lower temperature indicates a reduced degree of crystallinity, consistent with the higher UPy content. The increasing amorphous fraction in the series P1‐P2‐P3 is expected to improve the chain mobility with positive effects on the ion transport, as showed in the following sections [31, 32].

Tensile tests performed at 40°C (see Figure 3c) reveal a clear dependence of the mechanical strength from the supramolecular structure of the samples. At a fixed elongation of 0.6%, P1‐PEO exhibits the highest tensile strength (0.38 MPa), followed by P2‐PEO (0.28 MPa) and P3 (0.26 MPa). This progressive decrease of mechanical resistance reflects the transition from a more ordered and crystalline PE‐HEMA‐UPy network in P1 to a more amorphous backbone in P3. The increased amount of bulky UPy units moving from P1 to P3 reduces the crystallinity of the copolymer by hindering the crystalline packing of the PE segment. This behavior has been already observed in the pristine PE‐HEMA copolymer, in which the % of crystallinity decreases linearly as function of the amount of HEMA comonomer [21]. While the more amorphous character of P3 results in higher chain mobility and lower load‐bearing capacity [33, 34], P2 shows correspondingly moderate mechanical performance, as it is characterized by an intermediate level of supramolecular order.

The frequency‐sweep measurements, reported in Figure 3d, provide further insights into the viscoelastic behavior at 40°C. The storage modulus (E′), which represents the elastic component of the response, remains high across the entire frequency range (0.1–30 Hz) for P1‐PEO confirming its more rigid structure. P2‐PEO and P3‐PEO display lower E′ values, consistent with a random arrangement of molecular chains and an increased amorphous fraction, leading to a more flexible structure. The loss modulus (E″), indicative of viscous energy dissipation, follows a similar trend but with slightly smaller differences among the samples, suggesting that the main variation arises from the elastic contribution rather than the viscous one.

Notably, the frequency dependence of E′ is relatively weak for all samples, indicating stable network dynamics and limited relaxation processes within the probed frequency window. This behavior suggests that the supramolecular interactions remain active and effective at 40°C, maintaining structural integrity even under oscillatory deformation [35]. Taking in consideration the P3‐PEO separator, as illustrated in Figure 3e, f, depicting the stress‐strain profiles and the frequency behaviour response of the storage (E′) and loss (E″) moduli, the self‐healed specimen exhibits a marginal enhancement in tensile strength and mechanical properties closely matching those of the pristine counterparts, particularly in terms of their viscoelastic response.This behavior is ascribed to the re‐establishment of intermolecular hydrogen bonding between the UPy moieties during the healing process. Beyond the visual closure of the notch, these measurements provide direct evidence that the mechanical function of the membrane is restored: the healed specimen recovers the tensile strength of the pristine one and reproduces its storage and loss moduli over the whole probed frequency range, a healing efficiency close to unity in terms of both elastic and dissipative response.

#### 2.2.4 Functional Properties of Electrolyte‐Impregnated Self‐Healing Separators

**FIGURE 4:**
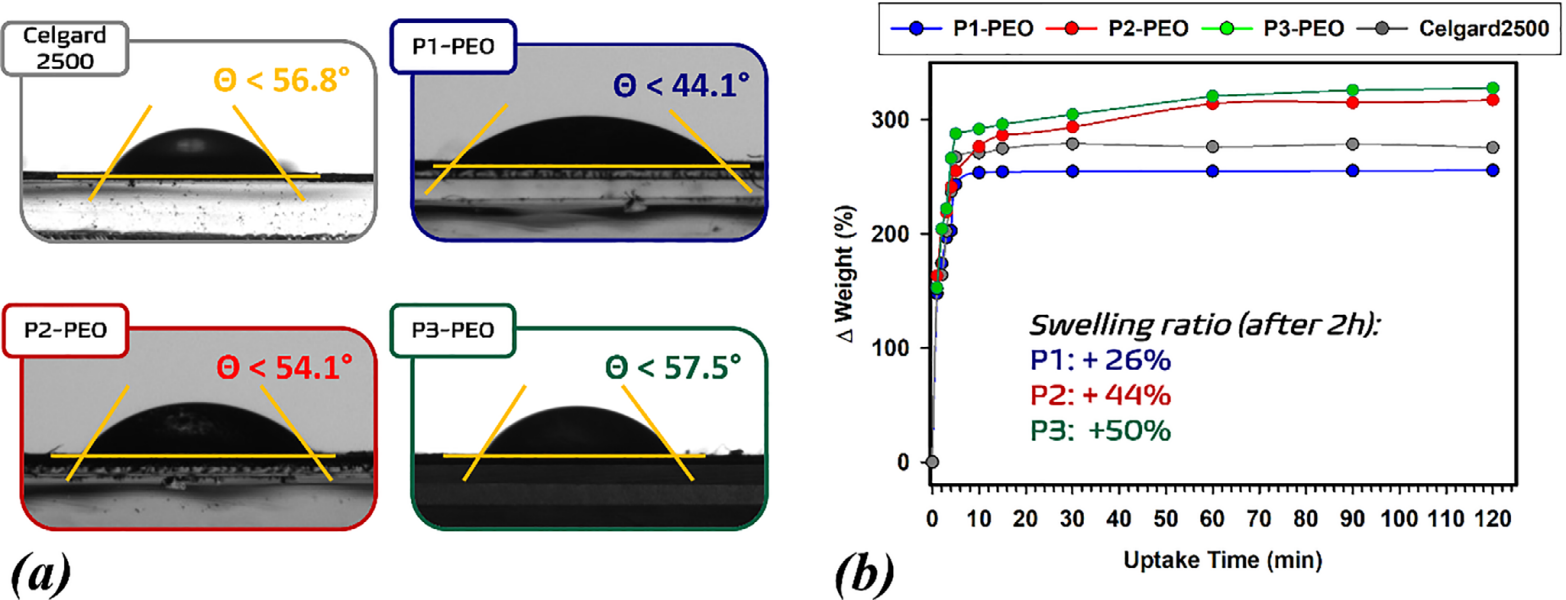
(a) Contact angle test with 1 M LiFSI in EC:DMC 50:50 as operating fluid, (b) the electrolyte uptake behavior. (The swelling ratio were calculated the following formula: $Swelling\;ratio:\;\frac{t_{1}-t_{0}}{t_{0}}\;\times 100$, where t_0_ and t_1_ are the thickness of the separator before and after electrolyte swelling).

Figure 4 shows the wettability and electrolyte uptake properties of the P1‐PEO, P2‐PEO, and P3‐PEO membranes compared with a commercial Celgard2500 separator, using 1 M LiFSI (Lithium bis(fluorosulfonyl)imide in EC:DMC 50:50 (Ethylene Carbonate:Dimethyl Carbonate), as the liquid electrolyte. Overall, contact angle measurements (Figure 4a) reveal a marked enhancement in the wettability of the P1‐PEO membrane (θ < 44.1°) compared to the Celgard2500 (θ < 56.8°). In contrast, the P2 (θ < 54.1°) and P3 (θ < 57.5°) membranes exhibit higher surface affinities toward the electrolyte that are comparable to those of the commercial reference.

In contrast, higher electrolyte uptake is observed in case of more amorphous separators. As shown in Figure 4b, after a rapid electrolyte absorption occurring within the first 20 minutes, the weight increase and the resulting swelling ratio follow the order P3‐PEO (+50%) > P2‐PEO (+44%) > P1‐PEO (+26%). The reason is that extended amorphous domains within the polymers could result in larger free volume and facilitate the solvent uptake [36, 37].

**FIGURE 5:**
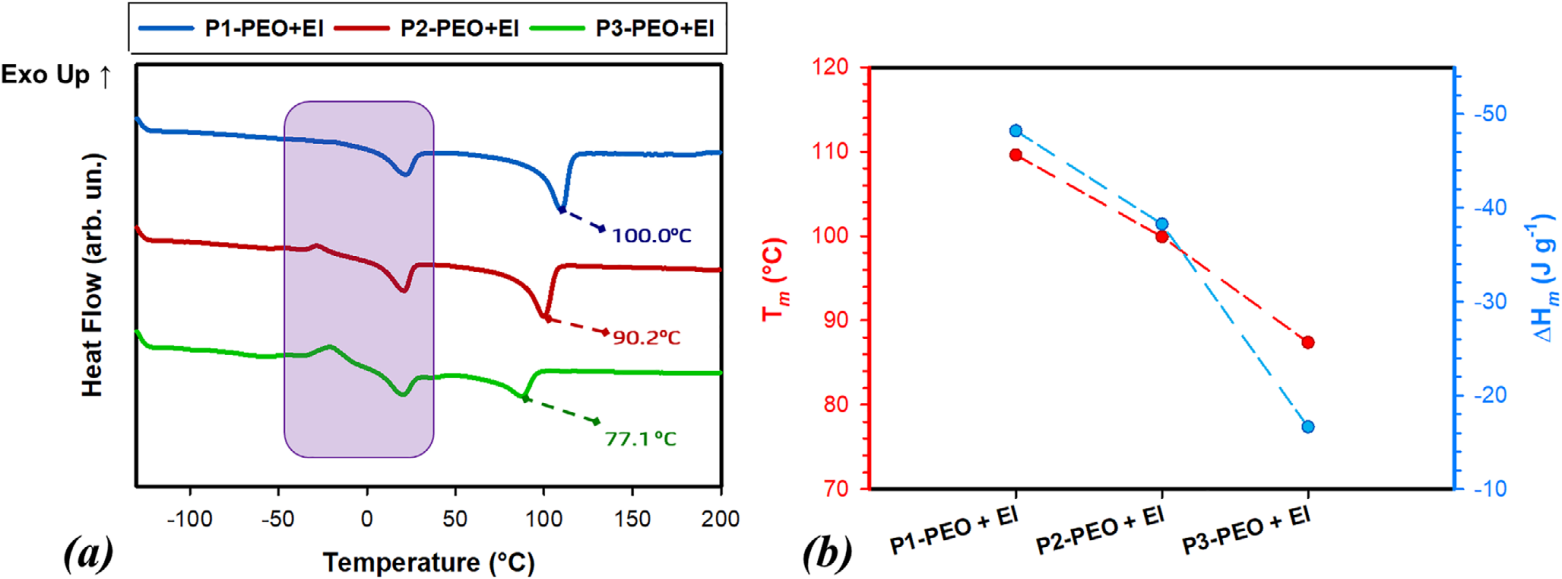
(a) DSC thermograms of the electrolyte‐activated PE‐HEMA‐UPy‐based separators. (b) Melting temperature (T_m,_ in red) and melting enthalpy (ΔH_m,_ in blue) of the composite self‐healing separators (P1‐PEO, P2‐PEO, and P3‐PEO) after electrolyte uptake.

Upon electrolyte uptake (referred as “+El”), the DSC curves (Figure 5a) reveal a clear modification of the separator thermal response. A broad endothermic feature appears around 20°C, corresponding to PEO‐electrolyte interactions associated with solvent coordination and partial disruption of PEO crystalline domains. In addition, the main melting transition of the PE‐HEMA‐UPy network shifts to lower temperatures and exhibits a significant reduction of the melting enthalpy compared to the pristine samples. Specifically, as reported in Figure 5b and Table S1, the melting temperatures (T*_m_*) of the PE‐HEMA‐UPy domains slightly shift upon electrolyte uptake, occurring at 109.6°C (P1‐PEO+El), 99.9°C (P2‐PEO+El), and 87.4°C (P3‐PEO+El). In contrast to the expected plasticizing effect, the melting enthalpies (ΔH*_m_*) increase compared to the pristine membranes, reaching −48.23 J g^−^^1^, −38.26 J g^−^^1^, and −16.65 J g^−^^1^ for P1‐PEO, P2‐PEO, and P3‐PEO, respectively. This behavior contrasts with the expected plasticizing effect, which would normally reduce crystallinity and melting enthalpy. We can speculate that the electrolyte induces a microphase reorganization. By preferentially solvating the amorphous regions, it enhances local chain mobility and promotes the rearrangement of polymer segments into more ordered configurations, resulting in an increased dimensional stability of the PE‐HEMA‐UPy crystalline regions.

**FIGURE 6:**
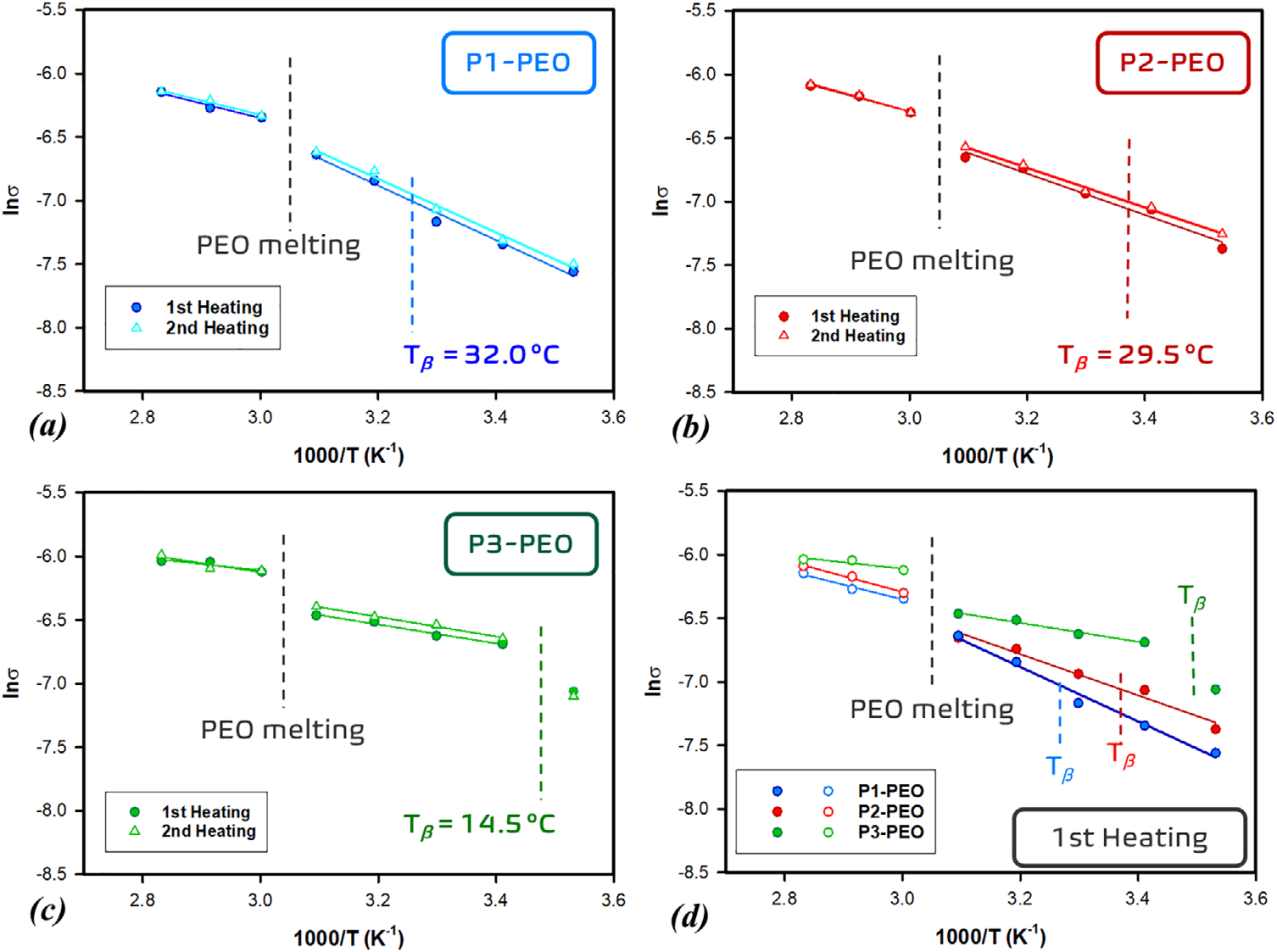
Ionic conductivity behavior as a function of temperature in the range 30  C–85°C for (a) P1‐PEO, (b) P2‐PEO, and (c) P3‐PEO for two heating cycles. (d) Comparison between the first heating cycle for all the investigated PE‐HEMA‐UPy – based separators separators.

Overall, the ionic transport behavior is in very nice agreement with the electrolyte uptake and amorphous fraction of the separators. High conductivity, whose value exceeds 1.5 mS cm^−1^ at 20°C is observed particularly in case of the P3‐PEO. The transport properties of the three polymer systems P1‐PEO, P2‐PEO, and P3‐PEO were evaluated through Arrhenius plots of ln σ versus 1000/T during the first and second heating cycles (Figure 6a–c). All samples display two distinct thermal regimes characterized by different slopes, below and above the melting transition of the PEO phase, respectively, as highlighted by the grey vertical dashed line. This discontinuity reflects the transition from a semi‐crystalline state, where restricted chain mobility hampers ion diffusion, to a more amorphous domain in which enhanced segmental dynamics markedly facilitate ionic transport. For systems P1‐PEO and P2‐PEO (Figure 6a,b), the ionic conductivity increases steadily with temperature, exhibiting comparable values in the high‐temperature region. The change in slope across the PEO melting point indicates a reduction in activation energy once the crystalline domains collapse and the polymer chains gain conformational freedom [38]. The beta transition temperatures (T*_β_*), extracted from the curvature at low temperatures, are 32.0°C for P1 and 29.5°C for P2, suggesting that the two matrices possess similar thermomechanical characteristics, with P2 exhibiting slightly enhanced segmental mobility. The P3‐PEO separator (Figure 6c), in contrast, shows a markedly different behavior. Its significantly lower T*_β_* (14.5°C) indicates a substantially more flexible polymer matrix, consistent with increased chain mobility even at moderate temperatures. A direct comparison of the three materials during the first heating cycle (Figure 6d) further highlights these trends: below the PEO melting point, P3‐PEO exhibits significantly higher conductivity values, attributable to its lower T*_β_* and enhanced amorphous character. Above the melting transition, the differences between the systems diminish, indicating that ionic transport is dominated by the overall segmental dynamics of the polymer matrix, which become similar across the three compositions. Both P1‐PEO and P2‐PEO exhibit relatively similar AE_1_ and AE_2_ values. In contrast, P3‐PEO displays markedly lower activation energies in both temperature segments, consistent with its more amorphous and flexible matrix (see Table S1). The conductivity determined during the second heating ramp show slightly higher values, as expected by considering the polymer chains rearrangement of PEO upon melting (see Figure S5 and Table S2) [39].

A specific comment is needed on the origin of the conductivity enhancement, that is, on whether it simply mirrors the larger amount of absorbed electrolyte or whether it reflects a genuine modification of the polymer microstructure. Two arguments support the latter interpretation. First, the two quantities do not scale with each other: the electrolyte uptake increases by a factor of about 1.9 on going form P1‐PEO (+26%) to P3‐PEO (+50%), whereas below the melting point of the PEO phase the ionic conductivity of P3‐PEO exceeds that of P1‐PEO by more than one order of magnitude (see Figure 6d). A purely additive contribution of the imbibed liquid phase would produce a proportional, and not exponential, effect. More importantly, the activation energy of the transport process is composition‐dependent: AE_1_ decreases form 0.184 eV (P1‐PEO) to 0.148 eV (P2‐PEO) and 0.067 eV (P3‐PEO), and AE_2_ follows the same trend (reported in Table S2). If the conduction occurred only within the liquid electrolyte confined in the membrane, the transport mechanism, and hence the activation energy, would be essentially the same in the three separators, and only the pre‐exponential factor, the number of carriers, would change. The observed decrease of the activation energy therefore indicates that the ionic motion is coupled to the segmental dynamics of the polymer host. This conclusion is corroborated by the quantitative correspondence between the T*_β_* values extracted from the change of slope of the conductivity plots and those independently determined by DMA on pristine membranes (32.0, 29.5, and 14.5°C for P1‐PEO, P2‐PEO, and P3‐PEO), reported in Figure 3b. The temperature at which the transport regime changes are dictated by the relaxation of the polymer matrix and not by the liquid phase, which is identical in the three systems. Another important, aspect obtained by DSC analysis of the soaked separators (see Figure 5) shows that the electrolyte uptake is not purely physical filling of the pores but induces a microphase reorganization of the polymer, with a shift of the melting transition and a redistribution of the crystalline fraction. The improved ionic conductivity is therefore ascribed to the combination of a larger amorphous, electrolyte‐swollen fraction and of a faster segmental dynamic of the host matrix, the electrolyte uptake being a consequence of the same microstructural evolution rather than an independent variable.

**FIGURE 7:**
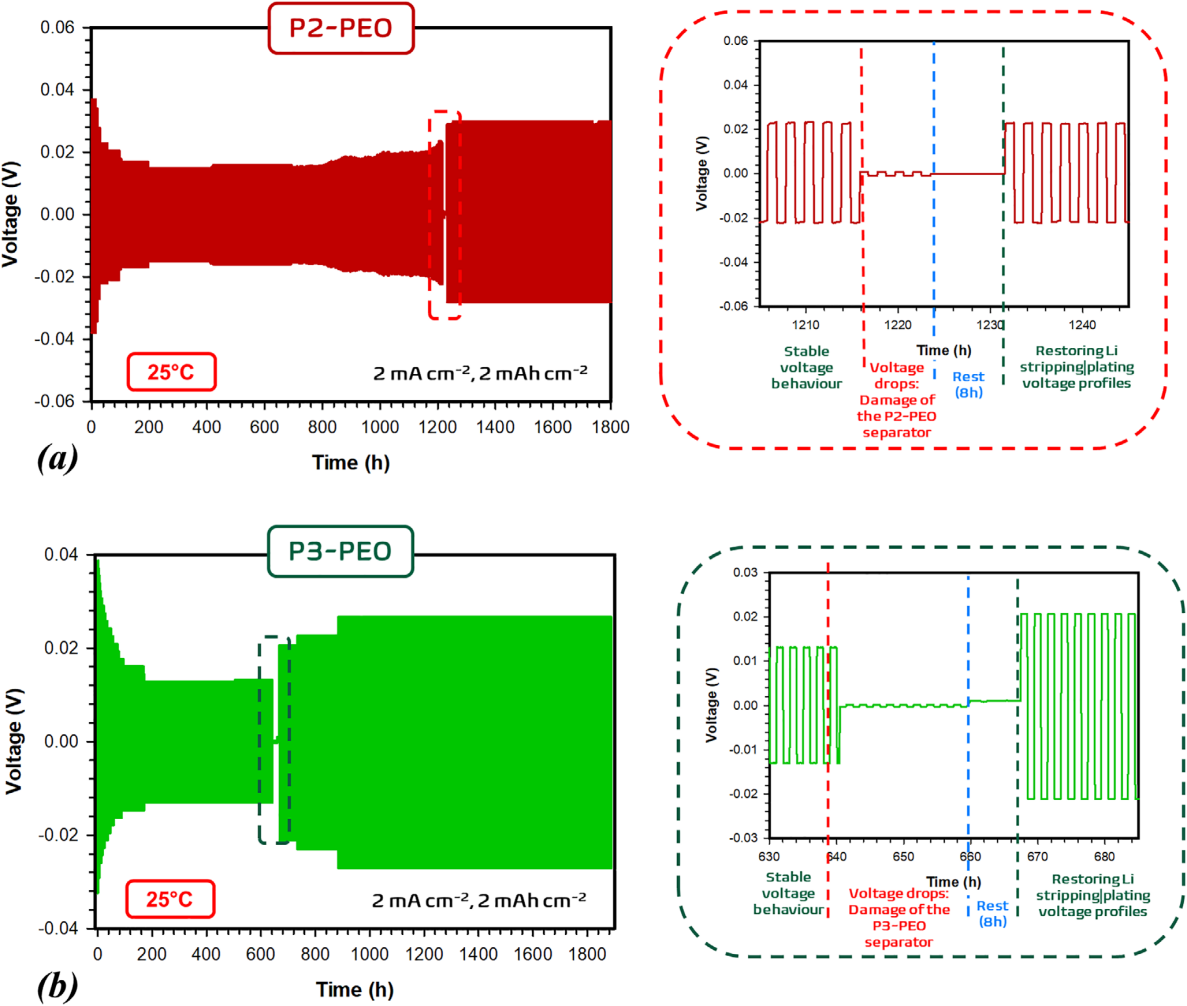
Galvanostatic stripping/plating tests performed on symmetric Li|SHS|Li cells at 25°C and a current density of 2 mA h cm^−^^2^. The figure shows the full cycling profile together with a magnified view of the cycles around the short‐circuit event. After the short circuit, an 8 h open‐circuit rest period was applied as a self‐healing step, after which the stripping/plating experiment was resumed. Results are reported for (a) P2‐PEO and (b) P3‐PEO, respectively.

To comprehensively evaluate the intrinsic safety and adaptive response of the supramolecular membranes under severe electrochemical stress, symmetric Li|separator|Li cells were subjected to prolonged galvanostatic stripping/plating tests combined with short‐circuit and open‐circuit rest protocols. This approach allows direct visualization of dendrite‐induced failure and, more importantly, assessment of the capability of the dynamic UPy‐based network to restore separator functionality after mechanically induced electrochemical damage. As shown in Figure 7 and Figure S6, a structure‐property correlation emerges when comparing the three SHS formulations with the commercial Celgard2500 benchmark. The Celgard separator (see Figure S6a) exhibits an initially regular polarization profile, followed by a progressive increase in overpotential and a sudden voltage collapse after 1350 cycles that ultimately marks irreversible cell failure due electrolyte depletion and decomposition. The P1‐PEO separator, however, undergoes electrodeposition instability after only 500 cycles, as reported in Figure S6b, resulting in enhanced polarization and voltage instability. In this case, the failure of the stripping/plating process is likely related to the separator microstructure discussed in the previous sections. Despite the presence of UPy motifs, the P1‐PEO separator is significantly more crystalline and prone to drying with respect to the other systems. This results in reduced solvent retention, and consequently a local electrolyte starvation that remarkably increase the cell interfacial resistance.

A significantly different scenario is observed for P2‐PEO and P3‐PEO (Figure 7). In these systems, long‐term cycling exhibits highly reproducible voltage oscillations and moderate polarization, that are indicative of a stable Li|separator interface and homogeneous ionic flux distribution. Upon the occurrence of a short‐circuit event, occurring after 1200 h for P2‐PEO and 640 h for P3‐PEO, the voltage transiently drops to near‐zero values, consistent with local dendritic bridging. However, after an 8 h open‐circuit rest, the stripping/plating profiles are restored, with voltage amplitudes and waveforms comparable to those recorded prior to failure. The magnified insets clearly illustrate the sequence of (i) stable operation, (ii) abrupt voltage drop due to separator damage, (iii) quiescent self‐healing stage, and (iv) re‐establishment of regular polarization cycles. This reversible behavior can be directly ascribed to the self‐healing capability of the separators taking place through the dynamic UPy‐UPy quadruple hydrogen‐bonding motifs embedded in the PE‐HEMA matrix, which enable supramolecular reorganization and closure of micro‐defects generated by lithium dendrites. It is worth noticing that the polarization amplitude recorded after the open‐circuit rest matches the pre‐failure values, and that the subsequent cycling proceeds with the same overpotential and waveform as before the short circuit event. Since the cell polarization is dominated by the ionic resistance of the electrolyte‐soaked separator and by the Li|separator interface, this observation demonstrates that the healing process restores the ionic transport across the membrane.

The evolution of the interfacial resistance and the lithium deposition morphology deserve a specific comment. In symmetric cell, the amplitude of the polarization and its evolution upon cycling are a direct result of the overall cell resistance and of the homogeneity of the lithium stripping/plating process. P2‐PEO and P3‐PEO display flat, symmetric, and reproducible voltage plateaus for hundreds of hours with a limited increase of overpotential (see Figure 7), which is the signature of a compact and uniform lithium deposition sustained by a homogeneous ionic flux, and of a stable, low‐resistance interphase. Conversely, the progressive rise of the overpotential observed for P1‐PEO after 500 cycles, together with the increasingly noisy voltage profile (reported in Figure S6b), point to a heterogeneous, dendritic lithium deposition promoted by a locally non‐uniform ionic flux, and to a continuous growth of the interfacial resistance caused by the local electrolyte starvation associated with the higher crystallinity and the lower electrolyte retention.

### 2.3 Electrochemical Performance of LFP‐Based Cells Including Self‐Healing Separators

**FIGURE 8:**
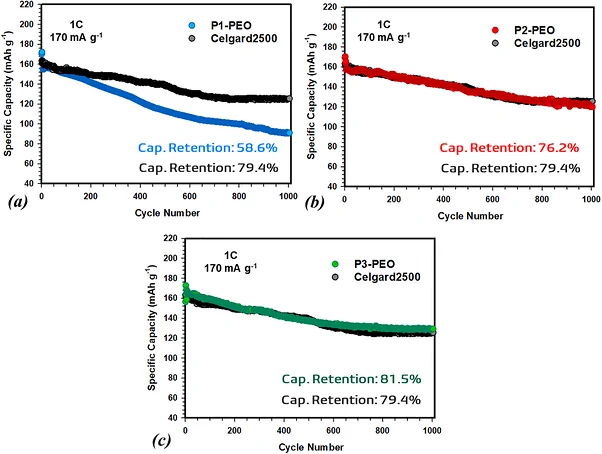
Long‐term cycling at 1C (1C: 170 mA g^−1^) of (a) P1‐PEO, (b) P2‐PEO, and (c) P3‐PEO separators two cells with 1M LiFSI in EC:DMC as liquid electrolyte. In all the figures is reported the discharge capacity values of a cell with commercial Celgard2500 separators (black‐grey circles) as benchmark.

Figure 8 shows the long‐term cycling performance of LFP‐based cells assembled with the three self‐healing polymer systems P1‐PEO, P2‐PEO, and P3‐PEO. The cycling performance was evaluated at a current rate of 1C (170 mA g^−1^), following an initial activation stage consisting of four low‐current cycles (two at 0.08C and two at 0.125C) to promote the formation of a stable SEI layer. In all cases, the corresponding performances were benchmarked against a cell employing a conventional Celgard2500.

As shown in Figure 8a, the P1‐PEO based cell exhibits a progressive decline in discharge capacity throughout cycling, leading to a capacity retention of 58.6% after 1000 cycles. This behavior suggests that the mechanical and interfacial stability provided by P1‐PEO is insufficient to sustain efficient lithium‐ion transport during extended operation, resulting in faster degradation in comparison to the benchmark separator (79.4%) [40, 41]. A markedly improved performance is instead observed for system P2‐PEO (Figure 8b). During the initial cycling regime, P2‐PEO delivers capacities comparable to the reference cell, and its capacity fading rate remains significantly lower at extended cycle numbers. After 1000 cycles, P2‐PEO retains 76.2% of its initial capacity. This enhancement indicates that the structural characteristics of P2‐PEO promote more favorable ion transport and interfacial stability during long‐term cycling. The best electrochemical performance is achieved with P3‐PEO separator (Figure 8c). Benefiting from its highly flexible polymer matrix and reduced T*_β_*, P3‐PEO exhibits reduced capacity decay over cycling, reaching a capacity retention of 81.5% with specific capacity higher than 130 mA h g^−1^ after 1000 cycles at 1C. As interesting result, the stabilizing effect of the more amorphous polymer environment mitigates interfacial resistance growth and facilitates robust Li^+^ transport during high‐rate cycling [42, 43]. It is evident from Figure S7 that all four cells investigated with the different separators (P1‐PEO, P2‐PEO, P3‐PEO, and the Celgard2500 reference) exhibit remarkably stable coulombic efficiencies exceeding 99% throughout the 1000‐cycle test, highly reversible Li^+^ intercalation/deintercalation processes and the absence of significant parasitic side reactions.

In further agreement with these results, Figure S8 reports the evolution of the galvanostatic voltage‐capacity profiles of the cells after 250, 500, 750, and 1000 cycles at 1C, highlighting the differences in polarization and kinetic stability induced by the various separators. The P1‐PEO system displays the least favorable behavior, with a progressive shortening of the charge/discharge plateaus and a pronounced increase in voltage hysteresis, indicative of a faster rise in internal resistance. A clear improvement is observed for P2‐PEO, whose profiles remain more stable and less distorted upon cycling. This tendency is further accentuated for P3‐PEO, particularly at advanced cycle numbers (750 and 1000), where the curves preserve a more regular shape and limited polarization, progressively approaching the profiles of the Celgard2500 benchmark.

### 2.4 Thermal Behavior of LFP‐Based Cells During Abuse Testing

**FIGURE 9:**
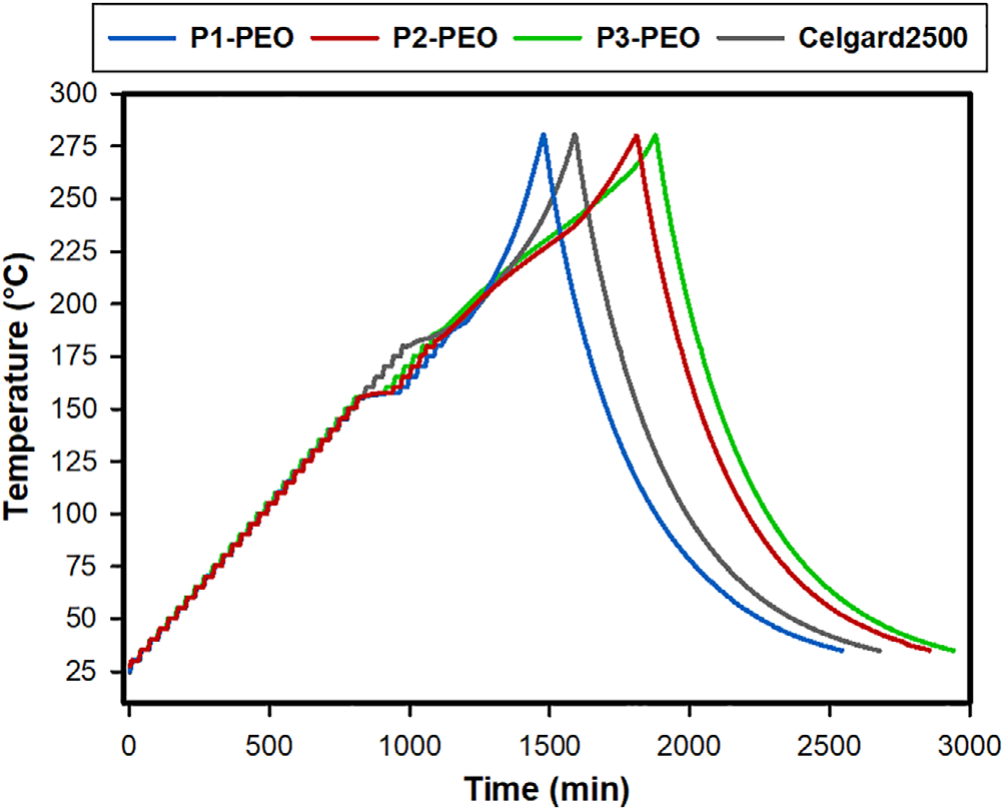
T vs t plots obtained by means of Accelerated Rate Calorimetry (ARC) performed on LFP|Li‐based cells including the P1‐PEO (blue line), P2‐PEO (red line), P3‐PEO (green line), and Celgard2500 (grey line), separators with 1M LiFSI in EC:DMC as liquid electrolyte.

The thermal stability of the PE‐HEMA‐based separators after electrolyte uptake was evaluated through Accelerating Rate Calorimetry (ARC) in a half‐cell configuration (LFP|Li) (see Figure 9). Determined and reported in Table 2, thermal parameters, including the onset temperature of the exothermal phenomena (T_Exo_), the total time to reach the maximum temperature (t_Max_), the maximum self‐heating rate (SHR_Max_), and average self‐heating rate (SHR_Average_) [44, 45]. As shown in Figure 9 and Table 2, the commercial Celgard2500 separator displays an exothermic onset at 180.2°C, with a SHR_Max_ of 0.488°C min^−1^ and a SHR_Average_ of 0.169°C min^−1^, consistent with its well‐known thermal shrinkage and subsequent electrolyte oxidation above 180°C.

**TABLE 2:** Thermal properties of the investigated electrolytes. T_Exo_, SHR_Max_, and SHR_Average_ onset temperature of the exothermal phenomena, maximum, and average self‐healing rate detected during the ARC overheating tests; tMax: time to trigger the thermal runaway in the cells.

Samples	T_Exo_ (°C)	t_MAX_ (min)	SHR_Max_ (°C·min^−1^)	SHR_Average_ (°C·min^−1^)
**Celgard**	180.2	1588	0.488	0.169
**P1‐PEO**	155.9 180.2	1475	0.037 0.555	0.0163 0.274
**P2‐PEO**	156.2 182.6	1808	0.039 0.385	0.0158 0.175
**P3‐PEO**	159.9 186.3	1876	0.038 0.300	0.0125 0.120

In contrast, the P1‐PEO separator, characterized by a higher crystalline fraction and lower UPy functionalization, exhibits exothermic events starting at lower temperatures compared to Celgard, namely at 155.9°C and 180.2°C, coupled to a SHR_Max_ of 0.555°C min^−1^ and a higher SHR_Average_ (0.274°C min^−1^). The enhanced crystallinity reduces the flexibility of the polymer network, while the limited supramolecular association provided by the lower UPy content decreases the ability of the matrix to absorb and redistribute thermal energy. Consequently, localized heat accumulation and electrolyte decomposition are promoted, leading to faster and more severe self‐heating once degradation initiates.

Conversely, P2‐PEO and P3‐PEO exhibit higher thermal stability and attenuated exothermic behavior, with onset temperatures shifted to 156.2/182.6°C and 159.9/186.3°C, respectively, and significantly lower average SHR values (0.175 and 0.120°C min^−1^). The larger hydrogen‐bonded UPy network and higher amorphous content enhance energy dissipation and limit the propagation of exothermic reactions, effectively improving the separator's thermal resilience.

The origin of the improved thermal response can be traced back to three concurrent contributions. (i) The intrinsic stability and the dimensional integrity of the membrane: the thermogravimetric analysis (see Figure S3) shows that the UPy‐functionalized PE‐HEMA network starts to degrade only above 380°C and that the blend are stable up to 350°C, well beyond the temperature range in which the polyolefin benchmark loses its integrity. Celgard2500 is a stretched, uniaxially oriented mesoporous film, whose stored orientation is released upon heating, causing a pronounced shrinkage, the direct contact between the electrodes and the sharp, self‐accelerating exotherm detected above 180°C. In the hot‐pressed SHSs, the physical cross‐links provided by UPy dimers restrain the chain mobility and, together with the absence of any residual orientation, limit the thermal shrinkage, so that the electrode separation, and hence the contribution of the internal short circuit to the heat generation, is preserved up to high temperatures. (ii) The dissociation of the quadruple hydrogen bonds is an endothermic, progressive process, so that the supramolecular network acts as a thermal buffer that absorbs and redistributes part of the heat released by the parasitic reactions. This is reflected in the mild, low‐rate exothermic event detected between 155.9 and 159.9°C, whose SHR_Max_ does not exceed 0.04°C min^−1^ (see Table 2): although it anticipates exothermic onset with respect to Celgard2500, this event dissipates part of the accumulated energy in a controlled way, without triggering the thermal runaway, and is followed by a main exotherm that is both delayed and markedly attenuated (t_Max_ up to 1876 min and SHR_Average_ as low as 0.120°C min‐1 for the P3‐PEO). (iii) The stronger interaction of the electrolyte with the amorphous, PEO‐rich domains, evidenced by the uptake measurements and by DSC of soaked membranes (reported in Figures 4 and 5, respectively), reduces the amount of free, highly volatile solvent available for the reaction with the delithiated cathode and with the lithium anode, and prevents the local electrolyte starvation and the consequent hot‐spot formation that instead affect the more crystalline P1‐PEO. The combination of these effects explain why the longest T_Max_ and the lowest SHR_Average_ are obtained for P3‐PEO, for the separator combining the highest UPy content, the largest amorphous fraction and the highest electrolyte retention, whereas P1‐PEO, in which the supramolecular network is more diluted and the matrix more crystalline, behaves worse than the commercial benchmark in term of self‐heating rate.

## 3 Conclusions

Supramolecular self‐healing systems based on UPy‐functionalized PE‐HEMA copolymers were successfully developed and evaluated as smart functional separators for lithium batteries. The incorporation of UPy motifs as reversible cross‐linking units in the PE‐HEMA copolymer generates a dynamic supramolecular network capable of autonomously repairing mechanical damage. By blending the PE‐HEMA‐UPy polymers with high‐molecular‐weight polyethylene oxide (PEO) and processing them through a rapid solvent‐free hot‐press method, robust and uniform composite separators were obtained with thickness and morphology suitable for battery applications.

The resulting membranes exhibit effective self‐healing behavior at mild temperatures, enabling the closure of mechanical defects through supramolecular reorganization. Structural tuning of the polymer composition strongly influences the balance between crystallinity, mechanical strength, electrolyte uptake, and ionic transport. Increasing the amorphous character of the polymer matrix improves segmental mobility and ionic conductivity while maintaining sufficient mechanical integrity. Among the investigated systems, the P3‐PEO separator demonstrates the most favorable electrochemical performance, delivering stable cycling in LFP‐based cells with capacity retention of 81.5% after 1000 cycles and excellent coulombic efficiency. Furthermore, abuse tests reveal enhanced thermal resilience compared with a commercial polyolefin separator.

Overall, this study demonstrates that the integration of dynamic supramolecular interactions into polyethylene‐based separator architecture provides an effective strategy to create adaptive, self‐healing membranes. Such materials offer a promising route toward safer and longer‐lasting lithium‐ batteries, and the design principles presented here may be extended to next‐generation multifunctional separators for advanced energy storage systems.

## Author Contributions

**Daniele Callegari**: investigation, writing – original draft, writing – review and editing, visualization, formal analysis, conceptualization, methodology, data curation, validation. **Arek Zych**: methodology. **Roberta Pinalli**: investigation, writing – review and editing, methodology. **Enrico Dalcanale**: conceptualization, investigation, writing – original draft, writing – review and editing, supervision, funding acquisition, visualization. **Eliana Quartarone**: funding acquisition, writing – review and editing, supervision.

## Conflicts of Interest

The authors declare no conflicts of interest.

## Supporting information




**Supporting File**: advs77850‐sup‐0001‐SuppMat.docx.

## Data Availability

The data that support the findings of this study are available in the Supporting Information of this article.
