# Supplementary material for: Self‐Healing UPy‐Functionalized Polyethylene Networks: A Robust Platform for Resilient and High‐Performance Lithium Battery Separators
Source: Adv Sci (Weinh). 2026 Sep 27:e77850. Online ahead of print. doi: 10.1002/advs.77850 (PMC13616283; doi:10.1002/advs.77850)
Supplement: Supplementary file 1 [file ADVS-9999-e77850-s001.docx]

**Supporting Information**

**Self-Healing UPy-Functionalized Polyethylene Networks: A Robust Platform for Resilient and High-Performance Lithium Battery Separators**

Daniele Callegari*^[a]^, Arek Zych^[b]^, Roberta Pinalli^[b]^, Enrico Dalcanale^[b]^, Eliana Quartarone^[a]^

[a] Department of Chemistry and INSTM-GISEL, University of Pavia, Via Taramelli 16, 27100 Pavia, Italy
E-Mail Corresponding author: [daniele.callegari@unipv.it](mailto:daniele.callegari@unipv.it)

[b] Department of Chemistry, Life Sciences and Environmental Sustainability and INSTM University of Parma, Parco Area delle Scienze, 17/A, 43124 Parma, Italy.

**Experimental Section**

P1-P2-P3 copolymers were prepared in a two-step synthesis according to published procedures (Scheme S1).^[19]^ The starting PE-HEMA copolymers, obtained via free radical copolymerization at high pressure in autoclave,^[21]^ are functionalized with UPy isocyanate to give the corresponding P1-P2-P3.

**Preparation of the Separators**

The Self-Healing Separators (SHSs) samples were prepared by a hot-press technique following a rapid and easily implementable solvent-free procedure (see Figure S1). In the first step, the self-healing polymer (PE-HEMA-UPy) was mechanically mixed with high–molecular-weight polyethylene oxide (PEO 300kDa) in a 2:1 weight-to-weight (w/w) ratio to enhance the electrolyte uptake capability. The resulting homogeneous blend was then subjected to hot pressing without the use of solvents, applying a temperature of 120 °C and a pressure of 400 psi for 10 minutes. After the thermomechanical treatment, uniform and free-standing polymer films were obtained, subsequently cooled to room temperature and used for further characterization. This methodology enables the fabrication of membranes with good structural integrity and improved affinity toward the electrolyte while maintaining a simple, reproducible, and environmentally friendly synthesis process.

**Cathode Preparation and Cell Assembly**

The cathode slurry was prepared by combining 80 wt% of LiFePO_4_ (LFP, MTI Corp.), 10 wt% of conductive carbon black (Imerys, Ensaco 350P), and 10 wt% of polyvinylidene fluoride (PVDF, Syensqo) dispersed in N-methyl pyrrolidone (NMP, Sigma-Aldrich 99.5%). The solid content of the slurry was 27 wt%. The cathodic active material, LFP, and carbon powders, were previously mixed in zirconia jars by a planetary ball miller at 150 rpm for 10 min twice, with a rest period of 5 min. Then, the polymeric binder was added and mixed with a similar procedure. After that, the resulting slurry was cast on a carbon-coated aluminium foil (MTI Corp.) using a doctor blade with 300 *μ*m wet thickness and then dried under vacuum at 40 °C. The cathode (active mass ~3 mg cm^-2^) was finally cut into 2 cm^-2^ disks and stored in a glove box (MBraun, H_2_O and O_2_*<*0.5 ppm) before the use. All the functional tests were performed using a 2032-type coin cell (MTI Corp.) assembled in the glove box. Metallic lithium (Sigma-Aldrich 99.9%, thickness 0.38 mm) was used as the anode. The same electrochemical tests were performed on control cells, including the 1M LiFSI in EC:DMC (50:50) solution (Solvionic) as the liquid electrolyte.

**Characterization**

Morphological characterizations were conducted using a SEC SNE-4500M Plus scanning electron microscope (SEM), operating under high vacuum at an accelerating voltage of 30 kV. Prior to imaging, all samples were coated with a thin carbon layer using a Cressington 208 carbon coater to ensure surface conductivity.

FTIR spectra of the self-healing separators (SHS) and of its precursors were collected using Spectrum Two (PerkinElmer), with a MIR DTGS (deuterated triglycine sulfate) detector, equipped with Universal Attenuated Total Reflectance (UATR) accessory. Air was used as the reference background. The spectra were obtained after pressing the samples on the ATR diamond crystal at room temperature, by adding four scans in the 4000-450 cm^-1^ range at 4 cm^-1^ resolution.

Elemental Analysis (EA). Elemental analyses were recorded on a CHNS Thermo Fisher FlashSmart. The nitrogen content was determined by means of thermal conductivity (TC) and volumetric analysis.

Thermogravimetric analyses of the precursor and realized separators were performed by means of a STA8000 (Perkin Elmer), by heating at 10 °C min^-1^ from room temperature up to 510 °C under a N_2_ atmosphere in a ceramic crucible.

The self-healing ability of the membranes was qualitatively assessed by a scalpel-cut test, and the scratched area was monitored by optical microscopy (Zeiss Olympia Axioplan).

Differential scanning calorimetry (DSC) was carried out by means of a DSC300 Caliris (Netzsch) in the temperature range between -130 °C and 200 °C at 10 °C min^-1^ under N_2_ atmosphere. Dynamic mechanical analysis (DMA) measurements were conducted on rectangular-shaped samples (7 mm×5 mm) using a DMA 242 E Artemis (Netzsch) equipped with a shear jaw for films. The beta transition temperature (T*_β_*) was measured by means of DMA at the frequency of 1 Hz from -80 °C to 40 °C with a heating ramp of 1K min^-1^. For the stress-strain investigation, the membranes were subjected to tensile testing at 40 °C with an increment strain of 0.01% up to the linear region limit. The frequency sweep tests were performed in the frequency range between 0.2 and 20 Hz at a temperature of 40 °C. Static contact angle analyses were performed to study the wettability of the different separators by means of a KSV CAM200 instrument with sessile drop method using 1M LiFSI in EC:DMC (50:50), (Solvionic) electrolyte as liquid medium. Additionally, all the separators were subjected to uptake testing with the same commercial electrolyte to assess any differences in electrolyte storage capacity. The membranes were soaked in liquid electrolyte and after the surplus solution was absorbed with paper, the separators were weighed periodically. The absorption ratio was measured by the following formula, where w_0_ and w_1_ are the masses of the separator before and after electrolyte immersion:

$$Electrolyte Uptake= \frac{w_{1}-w_{0}}{w_{0}} \times100$$

The ionic conductivity was measured between 10 and 80 °C via electrochemical impedance spectroscopy (EIS), by employing a Frequency Response Analyzer (Solartron 1255) connected to an Electrochemical Interface (Solartron 1287). An alternating current voltage of 50 mV was applied in the frequency range between 1 and 100 kHz.

The polarization experiment was studied by means of lithium stripping and plating test performed at 40 °C on a lithium symmetric coin cell, using an Arbin (model BT-2000) battery cycler. The cell was subjected to periodic cycling (1h per cycle) at a fixed current density of 2.0 mA cm^-2^.

Galvanostatic cycling with potential limitation (GCPL) was performed to assess the applicability of the SHS in a working cell. The coin cells were cycled on a Bio-Logic BCS-810 battery tester at room temperature in the voltage range of 2.4 to 3.8 V. Long-term stability tests were conducted at 1C (theoretical capacity of 170 mAh g^-1^), with two initial cycles at 0.2C, followed by two cycles at 0.5C. These initial steps were carried out to allow the SEI formation and stabilization before the long cycling test. All reported potentials refer to the Li^+^/Li couple.

Accelerated Rate Calorimetry (ARC-ES, THT) was used to assess the thermal stability of a half-cell in a thermal abuse test. The results of the SHS were compared with those of a conventional Celgard2500 separator, all soaked by the same electrolyte (1M LiFSI in EC:DMC (50:50), Solvionic). Before the ARC test, the coin cells with LFP cathodes were charged up to the limit voltage of 3.8 V, at a rate of 0.07 C and maintained at this voltage for 30 minutes. The accelerated rate calorimeter was operated in a heat-wait-search (HWS) mode with a sensitivity threshold detection limit of 0.02 °C min^-1^. The tests were performed in the temperature range of 30–280°C, with a stepwise increase of 5 °C, and 15-minute equilibration period. If the temperature change rate exceeded 0.02 °C min^-1^, the calorimeter would switch to “exotherm mode”, wherein self-heating occurs. If the rate were to exceed 1 °C min^-1^, the coin cell could potentially result in a thermal runaway.

**Supporting Information: Figures and Tables**

***Scheme S1****. Synthetic scheme for the preparation of P1, P2 and P3.*

***Figure S1.*** *Preparation steps of the PE-HEMA-UPy-based separators.*

***Figure S2.*** *Cross-sectional SEM images of (a)P1-PEO (38 μm), (b) P2-PEO (35 μm), and (c) P3-PEO (40 μm).*

***Figure S3.*** *Thermogravimetric analysis (TGA) plots of (a) precursors polymer and (b) the realized separators.*

| **SHS** | ***T_m_* (°C)** | ***ΔH_m_* (J g^-1^)** |
| --- | --- | --- |
| P1-PEO + Electrolyte | 109.6 | -48.23 |
| P2-PEO + Electrolyte | 99.9 | -38.26 |
| P3-PEO + Electrolyte | 87.4 | -16.65 |

***Table S1.*** *Melting temperature (T_m_) and enthalpy (ΔH_m_) of the composite self-healing separators (P1-PEO, P2-PEO, and P3-PEO) after electrolyte uptake.*

***Figure S4.*** *Optical microscopy images of the of PE-HEMA-PEO separator after (a) damage, (b) 3h, and (c) 18h at a temperature of 40 °C. No signs of self-healing were detectable.*

***Table S2.*** *Activation energy (AE) for the investigated separators imbedded with 1M LiFSI in EC:DMC as liquid electrolyte. AE_1_ and AE_2_ refers to the segments after the T_β_ and PEO melting, respectively.*

| ***First Heating Ramp*** | | |
| --- | --- | --- |
| ***Separator*** | ***Activation Energy AE_1_***  ***(after*** *T_β_****)*** | ***Activation Energy AE_2_***  ***(after PEO melting)*** |
| **P1-PEO** | 0.184 eV | 0.107 eV |
| **P2-PEO** | 0.148 eV | 0.100 eV |
| **P3-PEO** | 0.067 eV | 0.043 eV |
| ***Second Heating Ramp*** | | |
| ***Separator*** | ***Activation Energy AE_1_***  ***(after*** *T_β_****)*** | ***Activation Energy AE_2_***  ***(after PEO melting)*** |
| **P1-PEO** | 0.182 eV | 0.102 eV |
| **P2-PEO** | 0.131eV | 0.096 eV |
| **P3-PEO** | 0.064 eV | 0.041 eV |

***Figure S5.*** *Comparison between the second heating cycle for all the investigated PE-HEMA-UPy-based separators.*

***Figure S6.*** *Galvanostatic stripping and plating experiments on symmetric Li|Li cell at 25°C and a current density of 2 mAh cm^-2^ for (a) Celgard2500 and (b) P1-PEO.*

***Figure S7.*** *Specific capacity values and Coulombic Efficiency (%) for long-term stability tests of cells with (a) P1-PEO, (b) P2-PEO, (c) P3-PEO, and (d) Celgard2500 separators*

***Figure S8.*** *Galvanostatic charge-discharge voltage profiles of LFP half-cells assembled with (a)Celgard2500, (b)P1-PEO, (c) P2-PEO, and (d) P3-PEO separators, recorded at 1C (170 mA g⁻¹) after 250, 500, 750, and 1000 cycles.*

**UPy functionalization**

The degree of UPy functionalization was assessed by two independent methods: ^1^H NMR spectroscopy and nitrogen elemental analysis. In the NMR analysis, the UPy methyl and methylene resonances were integrated relative to the polyethylene backbone to determine the fraction of functionalized units (Figures S9-S11). Elemental analysis provided an independent estimate based on the nitrogen content, since nitrogen is present exclusively in the UPy pendant groups. The functionalization percentages were then calculated from the respective NMR integrations and measured nitrogen contents (Table S3).

***Figure S9.*** *Part of the ^1^H NMR spectrum of P1 in TCE-d2, 400 MHz, 393 K.*

***Figure S10****. Part of the ^1^H NMR spectrum of P2 in TCE-d2, 400 MHz, 393 K.*

***Figure S11.*** *Part of the ^1^H NMR spectrum of P3 in TCE-d2, 400 MHz, 393 K.*

***Table S3****. Theoretical and experimental C, H, N elemental analyses of P2 and P3 functionalized polymers.*

| ***Elemental Anlyses*** | %C | ***%H*** | ***%N*** |
| --- | --- | --- | --- |
| P2 65% Theoretical | 80,14%, | 13,14% | 1,08% |
| P2 65% Found | 83,66%**^a^** | 13,99%**^a^** | 1.07%; |
| P3 30% Theoretical | 77,72% | 12,56% | 2,45%; |
| P3 30% Found | 77,77% | 12,68% | 2,31% |

**^a^**The higher values of C and H found in P2 are due to residual toluene solvent present in the polymer
